# Molecular Insights into the Diversification and Biogeographic History of Six *Astragalus* L. Sections in the Turkish Flora

**DOI:** 10.3390/plants14142226

**Published:** 2025-07-18

**Authors:** Mevlüde Alev Ateş, Seher Karaman, Zeki Aytaç, Zeki Kaya

**Affiliations:** 1 Department of Agricultural Biotechnology, Faculty of Agriculture, Kırşehir Ahi Evran University, 40200 Kırşehir, Türkiye; 2 Department of Biological Sciences, Faculty of Science, Middle East Technical University, 06800 Ankara, Türkiye; 3 Department of Biology, Faculty of Art & Science, Aksaray University, 68100 Aksaray, Türkiye; seherkaraman@yahoo.com; 4 Department of Biology, Faculty of Science, Gazi University, 06000 Ankara, Türkiye; zaytac14@gmail.com

**Keywords:** *Astragalus*, cpDNA, nrDNA, phylogeny, molecular clock, biogeography

## Abstract

With 493 taxa and 63 sections, *Astragalus* L. is the largest genus in Türkiye. Most of these are narrow endemics and usually found in marginal habitats or require edaphic specializations (about 42% of the species are endemic). Due to the genus’s extensive diversity of species and common economic use, numerous scientific studies have concentrated on specific species. Taxonomic categorization based on morphological characteristics is insufficient to distinguish certain taxonomic groups. However, there is no systematic molecular phylogenetic analysis of Turkish species that deals with speciation in this genus. To concentrate on molecular-level speciation, fresh leaves from 152 samples representing 30 species across six sections native to Türkiye were collected over several months of comprehensive field studies and analyzed with regard to the internal transcribed spacer (ITS) of nrDNA and the *trn* L5′-L3′ + L3′-F(GAA) + *mat* K of cpDNA regions. Additionally, molecular clock estimations and biogeographical histories were analyzed to clearly understand the species’ divergence. Based on all studied regions, the *Poterion* section was found to be the newest and most divergent section, while the *Megalocystis* Bunge and *Halicacabus* Bunge sections were the closest and older ones. Furthermore, *A. vaginans* from section *Hymenocoleus* Bunge were included not only in this section but also in several other lineages. It is noteworthy that *A. dipodurus* and *A. oleaefolius* species from the section *Macrophyllium* Bunge are usually put together in a distinct sub-branch from other species members of the section in phylogenetic trees generated using both researched cpDNA and nrDNA regions. Moreover, some of the species are divided by the Anatolian diagonal, and the speciation of a significant number of species began during the Pleistocene geological time period. Geographical isolations or other weak isolation mechanisms preceded speciation in *Astragalus*, which requires more research in the future.

## 1. Introduction

*Astragalus* L. is a genus that has 3494 taxa, 746 of which are in the New World and 2748 in the Old World [1]. It is mostly widespread in Iran, Russia, and Türkiye [2]. Eurasia is often regarded as the continent of origin, with its center located in the Iran–Turan region according to the phytogeographic classification [3]. Volume 3 of the *Flora of Turkey* documents 372 species (391 taxa) [4], with 22 new taxa introduced in Volume 10 [5] and 23 new taxa in Volume 11 [6]. Recent systematist research has revealed that there are 493 taxa in 63 sections within the genus *Astragalus* in Türkiye [7,8,9,10,11,12,13,14,15,16,17,18,19,20,21,22,23,24,25,26].

The members of the genus are naturally found in steppe and mountainous regions of the Irano-Turanian Phytogeographic region of Türkiye. The great majority of endemic species are considered narrow endemics and are usually found in marginal habitats or require edaphic specializations [27]. Because of not only the high species diversity but also the economic importance of the genus, a number of studies dealing with diverse subjects of the genus have been carried out recently. The genus has a significant degree of variation in terms of fruit and vegetative morphology. For instance, in both the New and Old Worlds, leaf morphologies are frequently modified to respond to edaphic and climatic conditions [28]. Thus, *Astragalus* is considered a good example of adaptive radiation on a global scale, distributed generally in the northern hemisphere [29]. The taxonomy of *Astragalus* is complicated by these notable morphological variations, yet conventional taxonomic approaches are frequently unable to resolve the problems with this taxonomy.

Molecular methods are now frequently used to solve taxonomic problems in a variety of plant taxa [29]. In recent years, DNA barcoding techniques, which involve the analysis of standardized DNA markers, have demonstrated their utility for this purpose [30,31]. Kress et al. [32], Chen et al. [33], the China Plant BOL Group [34], and Kress [35] provide comprehensive accounts of the extensive application of these markers for species discrimination and the conservation of endangered species. The internal transcribed spacer (ITS) region of nuclear ribosomal DNA (nrDNA) has been extensively utilized in this context. The tRNA and *matK* gene regions on chloroplast DNA have recently been utilized as barcodes in plant identification. The simultaneous examination of cpDNA and nrDNA in a single study yields dependable outcomes for phylogenetic research [34]. Molecular data have proven valuable for elucidating evolutionary divergence time and patterns within the Fabaceae family, particularly the *Astragalus* genus [27,35,36], despite being restricted to a limited number of sections.

Compared with other flowering plants, the genus *Astragalus* exhibits a high degree of phylogenetic variety, according to earlier molecular phylogeny research on the genus [23,27,29,35,36,37,38,39,40,41,42,43,44]. DNA sequence data from nuclear or chloroplast gene regions were sufficient to identify phylogenetic links within the genus *Astragalus*; however, earlier research revealed that nuclear gene regions may be more reliable in interpreting evolutionary aspects of the genus due to their highly polymorphic nature. Thus, combining nuclear and chloroplast gene regions can provide more comprehensible information about which speciation was caused by climatic and topographic factors.

This study involved an extensive molecular analysis of various morphologically complex and phylogenetically close sections of the genus *Astragalus* in Türkiye; the *Macrophyllium* Bunge, *Hymenostegis* Bunge, *Poterion* Bunge, *Megalocystis* Bunge, *Halicacabus* Bunge, and *Hymenocoleus* Bunge. The taxa, which have historically presented challenges in taxonomic delineation due to overlapping morphological traits, were analyzed comparatively using both noncoding and coding regions of chloroplast DNA (*trn* L5′–L3′, *trn* L3′–F(GAA), and *matK*) alongside the internal transcribed spacer (ITS) region of nuclear ribosomal DNA. The work sought to elucidate evolutionary links and enhance comprehension of the molecular mechanisms underlying speciation within these sections by merging data from plastid and nuclear markers. Additionally, assessment of molecular divergence times and biogeographic analyses were conducted to delineate evolutionary time frames and geographic patterns of diversification.

## 2. Results

**Figure 1:**
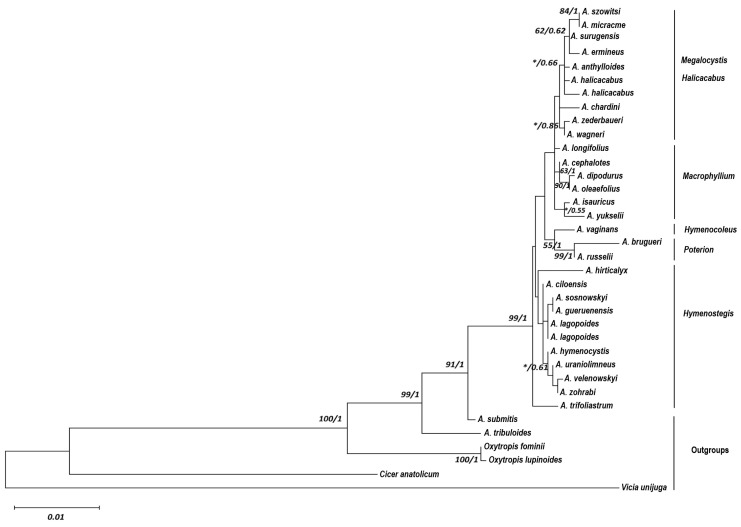
Phylogenetic tree with GTR model with Gamma distribution of cpDNA regions (*trn* L5′-L3′ + L3′-F(GAA) + *matK*) of studied species. The results of analysis with subsequent optimization (the posterior probabilities over 1 and bootstrap values with 1000 replicates) and values are given next to the nodes and separated with slashes (the bootstrap values lower than 50 and posterior probability values lower than 0.50 are not shown and indicated an asterisk (*)).

Combined sequence data analysis from three regions (*trn* L5′-L3′ + L3′-F(GAA) + *matK*) indicated that the total lengths of the regions were about 520 bp for trn L5′-F(GAA), 145 bp for trn L3′-F(GAA), and 1225 bp for *matK*). There was a total of 58 variable sites and 34 of these were found to be parsimony informative. The estimated total molecular diversity within a species was 0.007. The ITS region was about 615 bp in length with 49 variable sites, which were all parsimony-informative. Overall genetic diversity was calculated as 0.015 within the studied *Astragalus* species (Table 1). A phylogenetic tree was constructed based on the combined data from four cp DNA regions (*trn* L5′-L3′ + L3′-F(GAA) + *matK*), revealing that these gene regions supported section separations among studied *Astragalus* species (Figure 1). Generally, there were two main clusters composed of different sections. Those from sect. *Hymenostegis* (*A. ciloensis* Podlech, *A. lagopoides* Lam., *A. hymenocystis* Fisch.&C.A.Mey., *A. uraniolimneous* Boiss., *A. zohrabi* Bunge, *A. velenovskyi* Nábẽlek, *A. gueruenensis* Podlech, *A. sosnowskyi* Grossh., *A. hirticalyx* Bunge) were located in one of the main clusters, except *A. trifoliastrum* Hub.-Mor.&V.A.Matthews, and the others were located together; especially, sect. *Poterion* (*A. russelii* Banks&Sol. and *A. brugueri* Boiss.) and sect. *Hymenocoleous* (*A. vaginans* DC.) were positioned under same branch. The second major branch was composed of three sections and especially, sect. *Halicacabus* (*A. anthylloides* Pall., *A. zederbaueri* Stadlm., *A. halicacabus* Lam., *A. surugensis* Boiss.&Hausskn., *A. chardini* Boiss., *A. wagneri* Bunge) and sect. *Megalocystis* (*A. szowitsii* Fisch.&C.A.Mey., *A. micracme* Boiss.&Noë., *A. ermineus* V.A.Matthews) were clustered together. The other sect. *Macrophyllium* (*A. cephalotes* Banks&Sol., *A. dipodurus* Bunge, *A. oleaefolius* DC., *A. isauricus* Hub.-Mor.&V.A.Matthews, *A. longifolius* Lam.) were positioned under close branches from these two sections.

**Figure 2:**
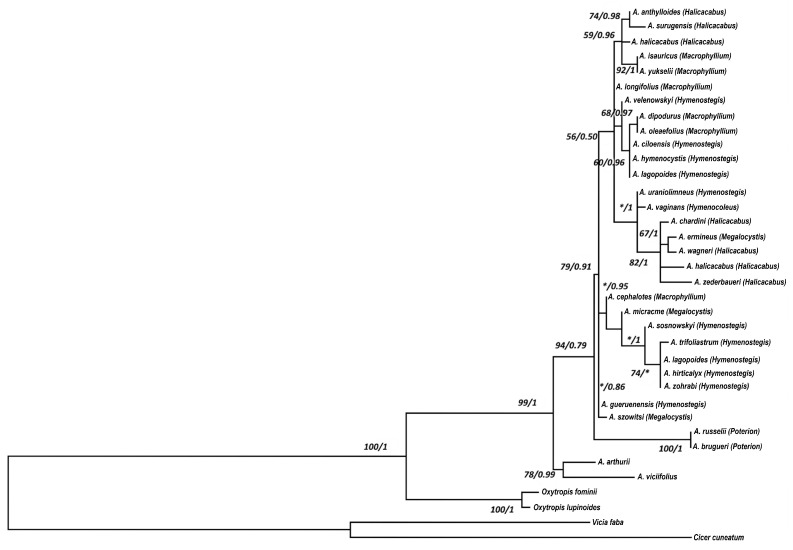
Phylogenetic tree with the GTR model with Gamma distribution of the nrDNA region (ITS) of studied species. The results of analysis with subsequent optimization (the posterior probabilities over 1 and bootstrap values with 1000 replicates) and values are given next to the nodes and separated with slashes (the bootstrap values lower than 50 and posterior probability values lower than 0.50 are not shown and indicated an asterisk (*)).

Additionally, data from the ITS regions of 30 species from six distinct *Astragalus* sections were used to form a molecular phylogenetic tree (Figure 2). Unlike the phylogenetic trees of combined cpDNA regions, the ITS tree did not indicate separations among sections. The phylogenetic tree divides *Astragalus* species into two primary clades. One clade is made up of sect. *Poterion* (*A. russelii* and *A. brugueri*), whereas the others are made up of different sections. *A. gueruenensis* of *Hymenostegis* and *A. szowitsii* of the *Megalocystis* sect are separated from others next to the base of the tree. Even if all other species are positioned differently from their section species, some of the separations and clusterings are parallel with the phylogenetic tree from the cpDNA regions. For example, *Macrophyllium* species are located together under the same branch, and they are separated from others in a different sub-cluster. On the other cluster, some morphologically similar species are arranged in nearby branches (for example, *Macrophyllium*; *A. dipodurus* and *A. oleaefolius*). As a result, while the distinctions at the section level in the phylogenetic tree based on the ITS region appear to be contradictory, the relationships among species are fundamentally consistent in many aspects with the species relationships determined through analyses of cpDNA regions (species with similar morphological traits). However, to understand the relationships of species more accurately, an extra phylogenetic tree was created using concatenated cpDNA and nrDNA regions, illustrating multiple clades within the genus *Astragalus*, signifying evolutionary divergence among the examined species (Supplementary Figure S1). The relationships among the sections are effectively seen in the tree, similar to the cpDNA-based phylogeny. In contrast to the cpDNA tree, the analysis of the combined dataset positioned *A. vaginans*, a member of section *Hymenocoleus*, into the same clade as species from section *Hymenostegis*. The outgroups *Oxytropis fominii* and *O. lupinoides* are distinctly separated from *Astragalus*.

**Figure 3:**
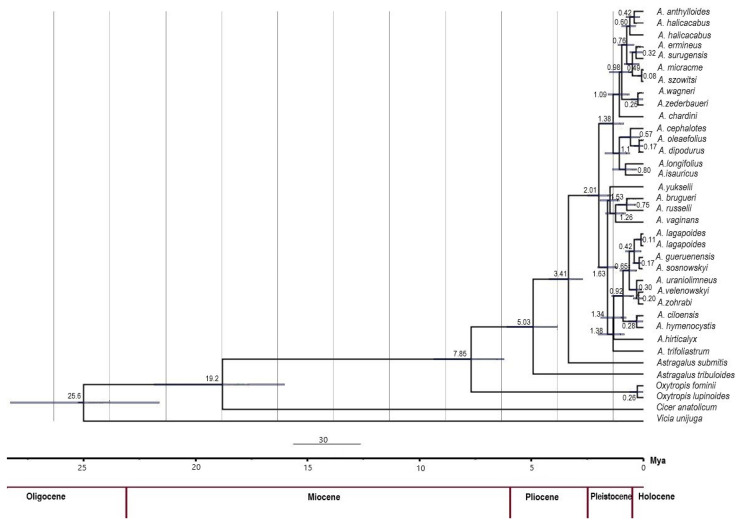
Ultrametric time-calibrated phylogenetic tree of 30 *Astragalus* taxa inferred using studied cpDNA regions. The analysis was conducted in the BEAST program under a relaxed log-normal clock model and Yule tree prior. The tree was summarized as a maximum clade credibility (MCC) tree using TreeAnnotator and visualized with FigTree v1.4.4. The divergence times are shown in million years (Mya), with the timeline progressing from the root (~30 Mya) to the present (0 Mya).

The Bayesian time-calibrated phylogenetic tree constructed using combined plastid markers (*trn*L-F + *matK*) revealed clear lineage differentiation among the 30 *Astragalus* taxa analyzed (Figure 3). The results suggest that six sections diverged from each other in the Pleistocene. Although most of the *Astragalus* species seemed to diverge from each other in the Pleistocene period, species within the *Hymenostegis* section appear to have diverged newly in the Holocene period (~0.6 MYA) from each other. Moreover, when it was checked at the species level, *A. micracme* and *A. szowitsi*, *A. dipodurus* and *A. oleaefolius*, *A. gueruenensis* and *A. sosnowskyi* newly diverged from each other during the Holocene period (~0.2 MYA).

**Figure 4:**
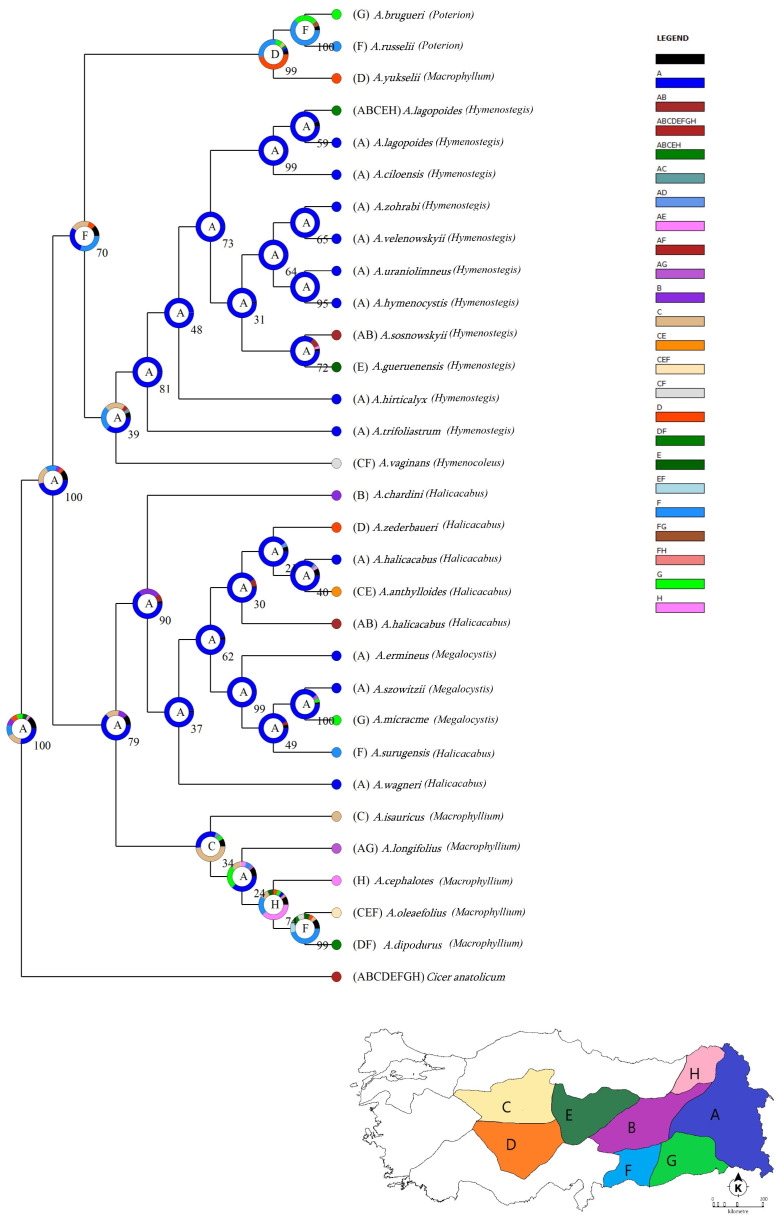
Tree output of S-DIVA analysis with MCMC runs (RASP program). Posterior probability values (PP) are shown over 100 and the most probable regions are shown with letters. Color legend refers to possible ancestral ranges at each node and biogeographical regions on the Türkiye map.

Additionally, RASP (Reconstruct Ancestral State in Phylogenies) analysis was conducted using a combination of studied cpDNA sequences. Based on the constructed phylogenetic analysis, the tree topology was indicated in the classification of six sections (Figure 4). Although the results were compatible with phylogenetic trees of cpDNA, only *A. vaginans* (sect. *Hymenocoleus*) were located under the same clade as the sect. *Hymenostegis* species. S-DIVA suggested a detailed biogeographical history where dispersal and vicariance played a significant role in species divergence of the current systematic sections of the *Astragalus* genus. In order to illustrate the spatial influences that led to speciation, geographic regions were identified in relation to the native distribution of species where samples were obtained by Dr. Karaman. For proper separation, different letters were given for each region: “A” for the east part of the East Anatolian Region, “B” for the west part of the East Anatolian Region, “C” for the Middle part of the Middle Anatolian Region, “D” for the south part of Middle Anatolian Region, “E” for the east part of the Middle Anatolian Region, “F” for west part of the South-East Anatolian Region, “G” for the east part of South-East Anatolian Region and “H” for the east part of the Black Sea Region (Figure 4). After analysis, it seemed that there were two main clades according to the RASP tree. One of the main clades was composed of three sections, and all *Macrophyllium* section species formed a different subclade except *A. yukselii* Karaman&Aytaç. This means that the *Macrophyllium* section was separated with a Bayesian credibility value of 34, and C was the most probable region of the species’ divergence region. Furthermore, species of both the *Halicacabus* and *Megalocystis* sections were positioned together with a value of 90, and the most probable divergence region was A. The other main cluster was composed of three different sections, and like the phylogenetic trees of cpDNA, the *Poterion* section and *A. yukselii* were located with a value of 70 and diverged in the F region. Interestingly, the only species of *Hymenocoleous* section *A. vaginans* was positioned with the species of *Hymenostegis*; however, it diverged at the same location with different values (39).

## 3. Discussion

In this study, six different sections of *Astragalus* species native to Türkiye were carefully sampled and investigated utilizing genetic data. Both cpDNA (*trn* L5′-L3′ + L3′-F(GAA) + *matK*) and nrDNA (ITS1 + 5.8S + ITS2) regions gave adequate variable sites to reveal genetic divergence among species and sections. Most of the variables’ sites were parsimony-informative and these were very useful for analysis. As a result of the large number of variable sites, speciation within the *Astragalus* L. genus continues. Additional sections and gene areas would be used in future studies to conduct molecular evolutionary research. Nonetheless, this study’s findings reveal significant molecular diversity data that offer insight into taxonomic and genetic links across six divisions of the *Astragalus* genus and species. Especially, interspecific diversity among *Astragalus* species that resulted in topographic positions gave significant data for molecular diversity studies on the genus.

Non-coding regions claim a faster evolution rate than coding regions. So, these non-coding regions give extensive data for phylogenetic studies. These regions are mostly used for interspecific connections and evolutionary relationships at various levels [45,46,47,48]. In the current study, the highest variable sites were found in the *trn* L-F region. Although this region was the shortest in length compared with other studied non-coding regions, it gave more informative data for the relationships of the studied sections of the genus. Shaw et al. [49] also reported that the *trn* L3′-F(GAA) region was much more diverse even if it was the shortest. According to earlier research [32,49,50], the *trn* L5′-L3′ region is not very good at differentiating closely related species and sections like *Astragalus*. The *matK* region has a specific gene encoding a maturase-like protein [51]. Although this coding region is longer than non-coding regions, low genetic divergence is expected because of its coding features. In the current study, the *matK* region had a low number of variable sites; therefore, it did not provide clear separation at the section level. To eliminate all these conflicts, all studied non-coding and coding cpDNA regions were combined and analyzed together. The analysis gave us more reliable results. Using this combination, a phylogenetic tree was constructed, showing a distinct differentiation, such as morphological separation at the section levels. All sect. *Hymenostegis* species except *A. trifoliastrum* were located on the same branch. Although *A. trifoliastrum* is a member of the sect. *Hymenostegis*, this separation reflects morphological differences in this species. Additionally, this result validated the molecular, morphological, and chromosomal revision of this species by Bagheri et al. [52]. Moreover, *A. trifoliastrum* could be studied alone from sect. *Hymenostegis*. In the main clade 2, there was a subclade composed of *A. vaginans* which is the only member of the sect. *Hymenocoleous*. Based on the morphological characteristics, Chamberlain and Matthews [4] stated in *Flora of Turkey* that *A. vaginans* is a distinct section from sect. *Hymenostegis*. Zarre and Podlech [53] reported that there were not adequate morphological differences to separate *A. vaginans* from the sect. *Hymenostegis*. However, Karaman Erkul et al. [54] indicated their agreement with previous taxonomic studies. Furthermore, Maassoumi [55]; Podlech et al. [56]; Bagheri et al. [57], and Podlech and Zarre [58] studied both morphological and molecular data, supporting Karaman Erkul’s study. Our results also support the placement of *A. vaginans* within the sect. *Hymenocoleous*.

Additionally, there was a subcluster composed of *Megalocystis, Halicacabus,* and *Macrophyllium* sections. Species of both *Halicacabus* and *Megalocystis* sections were located together based on a combination of studied cpDNA regions. Molecular and taxonomical studies indicated that morphological characters could not clarify infrageneric relationships in spiny and hairy *Astragalus* species [59]. After Maassoumi [55], Podlech [56], and Zarre [58], Nadari Safar et al. [59] determined that these two sections should be combined under the *Anthylloidei* section. In the current study, it was also revealed that species of these two sections are located together under the same branches based on cpDNA sequences. Currently, The *Flora of Turkey* is undergoing revision. The sections previously combined as *Anthylloidei* by Maassoumi [55] and Podlech and Zarre [58], are being re-separated into *Halicacabus* and *Megalocystis* in the newly written volumes.

Moreover, *A. oleaefolius* and *A. dipodurus* from sect. *Macrophllylium* were positioned very close to each other and although they were located under the same branch with other section members, they formed a single subbranch based on their genetic similarities. Zarre [60] indicated that although these two species from sect. *Macrophyllium* were morphologically similar to each other, they were slightly distinct from other members of the sect. *Macrophyllum* with strongly appressed leaf hairs in the former and spreading in the latter. Therefore, it can be safe to say that our molecular data is supported by morphological clues.

The other main cluster contained a very distinct section, *Poterion*. Tietze [61] demonstrated that sect. *Poterion* exhibits numerous distinct morphological characters compared to other sections. Notably, its leaf hairs are white and short, extending even to the calyx. Furthermore, phylogenetic analysis based on cpDNA regions places sect. *Poterion* is in a well-supported, single sub-branch.

According to the RASP analysis with S-DIVA methods, *A. yukselii* is in the subcluster with sect. *Poterion*, which is a categorization by Karaman Erkul and Aytaç [15]. *A. yukselii* distinguishes itself from Sect. *Macrophyllium* species by spreading hairy leaflets, pilose bracts at the apex, and some other traits. However, by many close characteristics, *A. yukselii* was accepted as a member of the sect. *Macrophyllium*. According to RASP analysis, it was situated far from the sect. *Macrophyllium*, both ancestrally and biogeographically. Therefore, in future studies, this species could be studied with more samples and more gene regions with different analyses (Figure 4).

In addition to cp DNA regions, ITS from the nrDNA region was also studied to clarify the phylogenetic relationships of six sections of the genus *Astragalus*. In contrast to the phylogenetic tree based on the cpDNA region, the ITS region-based tree did not resolve the studied species at the section level (Figure 2). Previous studies on sect. *Hymenostegis*, Bageri et al. [62] discovered that species resolution within the section *Hymenostegis* is quite poor, since many species have identical ITS sequences, even when these species are morphologically distinct. However, in our study, there were critical separations that supported the cpDNA results. Moreover, a species of sect. *Poterion* formed a single subclade alone. Also, while *A. dipodurus* and *A. oleaefolius* from sect. *Macrophyllium* were located at the same subbranch and clustered with *Hymenostegis* section species, they were positioned under the same main clade as other section members. Even if some species have different positions, species of *Halicacabus* and *Megalocystis* section species are located close to each other like in the tree of cpDNA. Like the phylogenetic tree of cpDNA, *A. yukselii* from sect. *Macrophyllium* is located very close to *A. isauricus* from the same section. Karaman Erkul and Aytaç [15] compared *A. yukselii* with *A. isauricus* and they reported that they had different morphological features; however, these were only used for analyzing species divergence, not at the section level.

The additional phylogenetic analysis using concatenated cpDNA and nrDNA sequences demonstrated considerable molecular difference across the examined *Astragalus* species, indicating both ancient divergences and recent speciation events. Robustly supported clades indicate tight evolutionary affiliations, perhaps originating from a common ancestor that underwent restricted geographic spread or ecological diversification. The close grouping of *A. dipodurus* and *A. oleaefolius* (bootstrap value: 98) suggests recent divergence, either due to microhabitat specialization or allopatric isolation. The moderate to poor bootstrap support shown in other internal nodes may be ascribed to quick radiation events, insufficient lineage sorting, or historical hybridization, which are prevalent in speciose taxa such as *Astragalus*. The occurrence of recurring species (e.g., *A. halicacabacus, A. micracme*, *A. lagopoides*) throughout several clades may indicate cryptic speciation or unresolved taxonomic intricacies (Supplementary Figure S1).

Molecular dating analysis indicated that the majority of speciation events among the studied species occurred primarily during the late Pleistocene and early Holocene (approximately 2–0.5 MYA) (Figure 3). A study by Bagheri et al. [63] utilized molecular dating to determine that the *Astragalus* section *Hypoglottidei* DC. assemblage originated 3.62 (1.73–5.62) million years ago, with significant diversification events observed within the past two million years. Numerous animal groups have diverged during the last 0.5 to 1 million years. Throughout these geological epochs, steppe ecosystems, favored by the majority of *Astragalus* species, underwent cycles of contraction and expansion in alignment with Pleistocene climatic fluctuations. Consequently, these climatic changes likely drove plant diversification through population fragmentation, leading to allopatric speciation, particularly within the Irano-Turanian steppe region, the main diversity center of the genus *Astragalus* [36].

Additionally, S-DIVA analysis suggested the Eastern part of Anatolia as a possible ancestral range for the studied species with a 100% marginal probability from *Cicer anatolicum*, which is another genus member of the Fabaceae family. During the late Pleistocene, Anatolian Sea levels were more than 130 m lower, leading to the formation of land bridges [64]. Following this period, most plant species persisted across both sides of the Anatolian Diagonal; some subsequently recolonized Europe, while others dispersed into Syria, Iraq, and the Near East [65]. Thus, although most of the studied species diverged from both sides of the Anatolian diagonal, they mostly came from the east part of Anatolia, according to S-DIVA analysis. The diversification pattern shown in the study aligns with the intricate geography and biological diversity of Anatolia, especially in areas like the Taurus Mountains and the Anatolian Diagonal, recognized for serving as both refugia and dispersing corridors. The geographic characteristics presumably facilitated vicariance-driven speciation, explaining the observed genetic structure and lineage divergence.

## 4. Materials and Methods

### 4.1. Plant Materials

**Figure 5:**
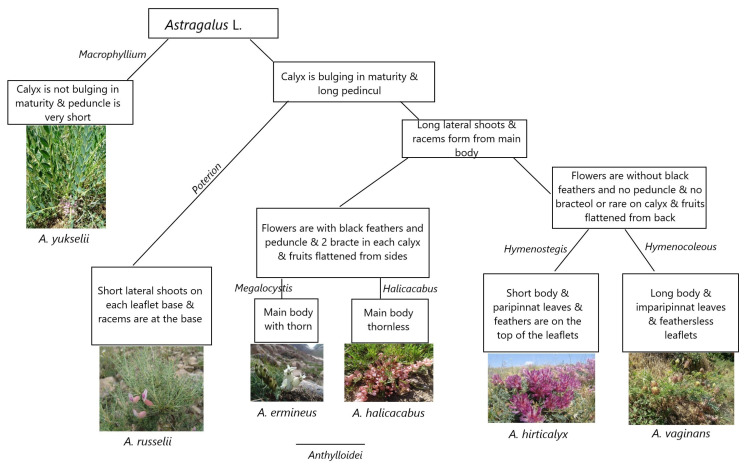
General morphological traits used as identification keys for six *Astragalus* L. sections studied in the current research.

**Figure 6:**
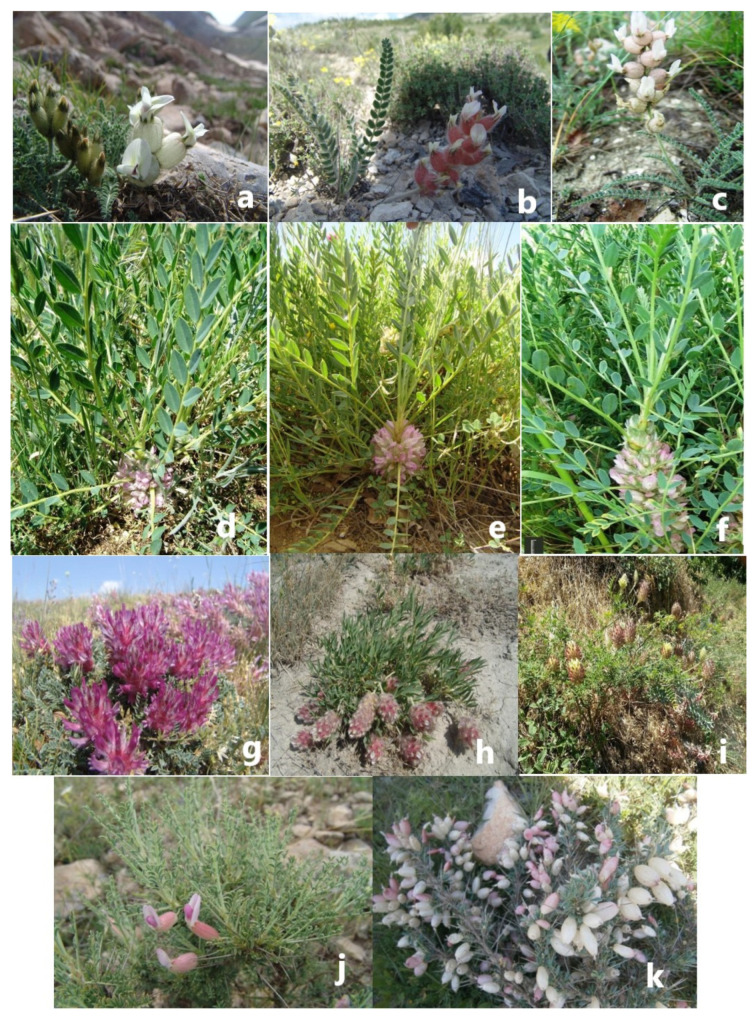
*Astragalus ermineus* (*Megalocystis*) (**a**), *A. zederbaueri* (*Halicabus*) (**b**), *A. anthylloides* (*Halicacabus*) (**c**), *A. oleaefolius* (*Macrophyllium*) (**d**), *A. isauricus* (*Macrophyllium*) (**e**), *A. yukselii* (*Macrophyllium*) (**f**), *A. hymenocystis* (*Hymenostegis*) (**g**), *A. trifoliastrum* (*Hymenostegis*) (**h**), *A. vaginans* (*Hymenocoleus*) (**i**), *A. russelii* (*Poterion*) (**j**), *A. brugueri* (*Poterion*) (**k**) flowers with specific structures (Photos: S.Karaman, 2014).

**Figure 7:**
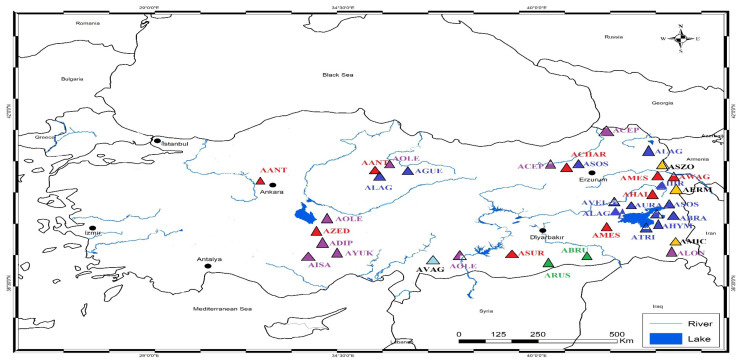
The Türkiye map shows the different locations of samples of *Astragalus* species in the study. The species are coded as AANT = *A. anthylloides*, ACHAR = *A. chardini*, AMES = *A. halicacabus*, AWAG = *A. wagneri*, AHAL= *A. halicacabus*, ASUR = *A. surugensis*, AZED = *A. zederbaueri*, AOLE = *A. oleaefolius*, ADIP = *A. dipodurus*, AISA = *A. isauricus*, AYUK = *A. yukselii*, ALON = *A. longifolius*, ACEP = *A. cephalotes*, AVAG = *A. vaginans*, ARUS = *A. russelii*, ABRU = *A. brugueri*, ASZO = *A. szowitsii*, AMIC = *A. micracme*, AERM = *A. ermineus*, ALAG = *A. lagopoides*, ASOS = *A. sosnowskyi*, AGUE = *A. gueruenensis*, AVEL = *A. velenowskyi*, ABRA = *A. lagopoides*, ATRI = *A. trifoliastrum*, AHYM = *A. hymenocystis*, AURA = *A. uraniolimneus*, AZOH = *A. zohrabi*, AHIR = *A. hirticalyx*, ASUR = *A. surugensis*, ACIL = *A. ciloensis*.

Fresh leaves of 152 individual plant samples representing 30 species belonging to the six sections of *Astragalus* L. (*Macrophyllium*, *Hymenostegis*, *Poterion*, *Megalocystis*, *Halicacabus*, and *Hymenocoleus* sections) were collected by Dr. Seher Karaman during her field studies conducted from 2011–2014 (Figure 5, Figure 6 and Figure 7). The number of samples per species varied from 3 to 8 depending on how a species is widespread or restricted natural distribution. Taxonomic identifications of species were carried out with extensive field and herbarium works. All samples were conserved at Prof. Dr. Tuna Ekim Herbarium (GAZI) and Aksaray University Herbarium (AKSU). Collection locations and voucher numbers are given in list Appendix A, Table A1.

### 4.2. DNA Extraction, Amplification, and Sequencing

152 samples representing 30 *Astragalus* L. species belonging to six sections that are native to Türkiye, were used to extract total DNA. In DNA extraction, a modified 2XCTAB (cetyltrimethylammonium bromide) protocol [66] was used.

For the amplification of *trn* L5′-L3′ + L3′-F(GAA) intergenic spacer regions of cpDNA, two primer pairs described in Taberlet et al. [67] were used. For the *matK* region, the primers described by Li et al. [68] were used. The primers for the region were sourced from the study to amplify the ITS region from Hsiao et al. [69] were chosen. The polymerase chain reactions (PCR) for all studied regions were performed with a total volume of 25 μL containing 12 μL 10mM dNTP mixture including 10Xbuffer and 25mM MgCl^2^, 0.25 μL each primer pair, 1 μL template DNA and 11.5 μL dd H_2_O in 0.2 mL sterile Eppendorf tubes.

PCRs were performed with a Thermocycler (Eppendorf^®^ Mastercycler^®^, HH, DE) by optimized cycling parameters as: initial denaturation at 94 °C for 5 min followed by 35 cycles at 95 °C for 30 s of denaturation, at 56 °C (*trn* L regions), 58 °C (*matK* & ITS region) and 60 °C (*trn* L-F region) for 30 s of annealing, at 72 °C for 90 s of extension, and at 72 °C for 10 min of final extension. The PCR products were visualized by electrophoresis in 1.5% agarose gel. The PCR products of amplification of target regions were stored at −20 °C until they were sent for sequencing. The purification and sequencing procedures were done by the RefGen Biotechnology facilities (Ankara University, Technocity, Gölbaşı, Ankara). An ABI3730XL 96 capillary automatic sequencer was used for sequencing amplified DNA products of nuclear and chloroplast gene regions.

### 4.3. Data Analysis

All sequences were checked and examined by Finch TV software (Version 1.4.0-manufactured by Geopiza Research Team, https://finchtv.software.informer.com/1.4/, accessed on 1 April 2025) [70] to eliminate ambiguous base calls before data analyses. Sequences were aligned by the MUSCLE (Multiple Sequence Comparison by Log Expectation) tool [71] of MEGA 11 (Molecular Evolutionary Genetics Analysis) software [72]. Molecular diversity statistics such as GC contents (%), nucleotide deletions and insertions, conserved and variable sites, parsimony informative sites, transition/transversion (tr/tv) ratio, and nucleotide diversity were calculated via MEGA 11 software for each chloroplast and nuclear gene region separately (Table 1). Moreover, the intra- and interspecific divergence among studied species and within sections were also calculated via the MEGA 11 program by using a combination of all studied gene regions (cpDNA and nrDNA), and the genetic divergence matrices were obtained (Supplementary Tables S1 and S2)

The optimum substitution model for reconstructing the species’ phylogeny was determined using the Model Test program with the MEGA 11 software. The computer program suggested utilizing the general time reversible (GTR) model with Gamma distribution for sequence analysis (based on AICs value) [73], and the phylogenetic trees were generated by employing the maximum likelihood method based on the GTR+G model, accompanied by bootstrap test analysis. This study utilized the Bayesian evolutionary analysis by sampling trees (BEAST) software tool, employing the GTR+G substitution model with uniform rates across data partitions. The Yule tree was utilized, and an initial tree was randomly produced by 10,000,000 Markov chain Monte Carlo (MCMC) iterations. The raxmlGUI 2.0 tool (https://cme.h-its.org/exelixis/web/software/raxml/index.html, accessed on 2 April 2025) was employed, with the GTR+fast bootstrap values method, to verify and confirm the reliability of the data produced. The phylogenetic complex trees were further condensed and amalgamated using the Tree Annotator software (https://beast.community/treeannotator, accessed on 8 April 2025), applying a posterior probability threshold of 1 [74,75]. The trees were shown using Fig Tree V 1.4.4 (http://tree.bio.ed.ac.uk/software/figtree/, accessed on 9 April 2025) [76]. The phylogenetic trees produced independently by the MEGA and BEAST software using the same model (GTR+G) had their bootstrap and posterior probability values manually combined into a single tree for comparative and integrative study. The sequences of the examined *Astragalus* species, along with selected and distinct genera like *Vicia unijuga* A. Braun (accession numbers *matK*: HM026402.1 & tRNA: KP699023.1) and *Vicia faba* L. (accession number: ITS: MW843838.1), *Cicer anatolicum* Alef. (accession numbers: *trn* AB117673.1 & *matK* AB198872.1), *Cicer cuneatum* Hochst. ex A. Rich. (ITS: MW424518.1), *Oxytropis fominii* Grossh. (accession numbers: *trn* KR908683.1; *matK* KM387608.1; ITS KM053390.1), *Oxytropis lupinoides* Grossh. (accession numbers: *trn* LC213516.1; *matK* KM387604.1; ITS LC213393.1), and various *Astragalus* species (accession numbers: *A. submitis* Boiss.& Hohen. *matK*: KX955194.1 & tRNA: AB485935.1, *A. tribuloides* Kotschy ex Bunge *matK*: KX955200.1 & tRNA: AB485929.1, *A. viciifolius* DC. ITS: JQ685639.1, *A. arthurii* M.E. Jones ITS: KC433896.1) were sourced from the NCBI database and utilized as outgroups. The sequences were included with the samples utilized in the analysis to provide an evolutionary context.

In the current study, a time-calibrated phylogenetic analysis was used with BEAST software, which facilitates Bayesian inference of DNA sequence data within time-structured evolutionary frameworks. BEAST integrates many frameworks, including phylodynamic models, divergence time estimates, spatial phylogeographic reconstruction, and trait evolution studies including both discrete and continuous variables [74]. The approach used merged cpDNA areas and employed a relaxed log-normal molecular clock model with a Yule speciation before addressing interspecific diversification. Two calibration priors were implemented: the initial one at the root node (i.e., *Astragalus* versus outgroup), constrained by a secondary calibration point of 25 ± 5 million years ago (Mya), derived from prior estimates within the Fabaceae family; the subsequent one at the crown node of *Astragalus*, constrained with a lognormal prior offset at 12 Mya, with a mean of 2.7 and a standard deviation of 0.4, mirroring earlier diversification estimates within the genus [27,36]. The investigation was conducted across 10 million MCMC generations, with convergence evaluated by Tracer (https://beast.community/tracer, accessed on 6 July 2025), confirming effective sample sizes (ESS) above 200. The ultimate maximum clade credibility (MCC) tree was compiled with Tree Annotator and shown in Fig Tree v1.4.4.

Further, historical biogeographical reconstruction analysis based on cpDNA phylogenetic data was carried out using the RASP (reconstruct ancestral state in phylogenies) program [77] which is very helpful for reconstructing evolutionary histories in phylogeny [78]. The options of S-DIVA (statistical DIVA), Bayesian binary MCMC, and maximum-parsimony (MP) analysis of RASP were selected to obtain ancestral probability ranges (%) at each node from each sampled region. In the RASP analysis, biogeographic regions were selected via collection areas, to indicate how speciation was affected by geographic barriers.

## 5. Conclusions

The molecular trees acquired in the current study (cpDNA and nrDNA) are incompatible in some ways. Generally, it has been known that molecular data do not necessarily agree perfectly with the actual evolutionary pathways of the taxa [79,80,81]. Many different processes result in this differentiation such as hybridization, introgression, lineage sorting, etc. The positioning of species may have resulted from hybridization events and subsequent chloroplast capture many years ago, particularly between the trees from nuclear and chloroplast DNA [29]. Moreover, it is questionable to balance the causes of genetic drift, inbreeding depression, and stochastic oscillations of the environment, due to the small number of individuals in a population [82]. Thus, more research is required to elucidate the function of natural hybridization in speciation. Furthermore, it is typical for environmental factors to influence morphological traits in addition to genes. The current study’s sample size appears sufficient to represent the molecular relationships among the studied sections. Although the studied species exhibited low genetic divergence, these data successfully distinguished species at the section level. Furthermore, cpDNA regions, particularly non-coding regions, proved more informative for phylogenetic resolution. Despite the nrDNA region not separating sections, key aspects of the phylogenetic tree’s topology largely supported the existing taxonomic classification.

In conclusion, our results emphasize the evolutionary intricacy of Turkish *Astragalus* lineages and identify Anatolia as a pivotal hub of diversification and endemism for the species. Subsequent research using genome-wide data and extensive regional sampling will be crucial for elucidating the phylogenetic linkages and evolutionary processes influencing this taxonomically diverse group. Furthermore, the incorporation of reproductive biology, population genetics, and divergence time studies will enhance the thoroughness and reliability of the categorization of essential *Astragalus* species.

## Figures and Tables

**Table 1:** Molecular diversity statistics for studying cpDNA and nrDNA regions at the MEGA 11 program (Kimura-2 statistical parameters).

	cp DNA			ITS (ITS1 + 5.8S + ITS2)
	*trn* L5′-L3′	*trn* L3′-F(GAA)	*matK*	
Number of species	30	30	30	30
Total length (bp)	520	145	1225	615
GC content (%)	30.5	34.1	31	53.9
Conserved sites	471	119	1188	553
Variable sites	20	15	23	49
Parsimony informative sites	12	15	7	49
Transitional pairs	40.14	43.87	42.27	60
Transversional pairs	59.86	56.13	57.73	30
Transition/transversion (tr/tv) (R)ratio	0.58	0.95	0.63	1.48
Molecular diversity	0.04	0.028	0.017	0.015

## Data Availability

The sequences that were used in the study were uploaded to the NCBI data base (https://www.ncbi.nlm.nih.gov/, uploaded at 9 July 2025) and Accession numbers are given in the Appendix A (Table A1).

## Supplementary Materials

The following supporting information can be downloaded at: https://www.mdpi.com/article/10.3390/plants14142226/s1, Figure S1: Phylogenetic tree of combination of both cpDNA regions (*trn* L5′-L3′ + L3′-F(GAA) + *matK*) and nrDNA regions (ITS) of the studied species; Table S1: The number of base substitutions per site from averaging all sequence pairs between species; Table S2: The number of base substitutions per site from averaging all sequence pairs between sections.

## Abbreviations

The following abbreviations are used in this manuscript:

NCBI	National Center for Biotechnology Information
MEGA	Molecular Evolutionary Genetic Analysis
BEAST	Bayesian Evolutionary Analysis Sampling Trees
RASP	Reconstruction of Ancestral State in Phylogenies
S-DIVA	Statistical DIVA
MCMC	Markov Chain Monte Carlo
AIC	Akaike Information Criterion

## Appendix A

**Table A1:** Geographical information and voucher numbers of the studied *Astragalus* L. species collected in field studies and GenBank numbers of the studied species (indicated with asterisk (*)) (ITS, *trn*L-F (*trnL5′*-L3′+ L3′-F(GAA)), *matK* respectively).

Section	Sample Number (SK)	Species Name	Location (Town/Province)	GenBank Accession Numbers
***Halicacabus***	2541, 2617	*A. anthylloides* *	Ankara/Ayaş	PV897526, PV902929, PV902899
	2558	*A. anthylloides*	Sivas/Divriği	
	2567	*A. chardini* *	Erzurum/Oltu	PV897528, PV902930, PV902900
	2571	*A. chardini*	Erzurum/Horasan	
	2578	*A. halicacabus* *	Van/Muradiye	PV897532, PV902931, PV902901
	2585	*A. halicacabus*	Van/Pirreşit Dağı	
	2701	*A. halicacabus*	Ağrı/Murat Vadisi	
	2704, 2713	*A. halicacabus*	Muş/Malazgirt	
	2706	*A. halicacabus* *	Van/Bahçesaray	PV897538, PV902932, PV902902
	2709	*A. halicacabus*	Van/Muradiye	
	2572	*A. wagneri* *	Ağrı/Doğubeyazıt	PV897547, PV902933, PV902903
	2543	*A. zederbaueri* *	Konya/Altınapa Barajı	PV897549, PV902927, PV902897
	2547	*A. zederbaueri*	Konya/Beyşehir Yolu	
	2548	*A. zederbaueri*	Konya/Ermenek	
	2697	*A. surugensis* *	Ş.Urfa/Hilvan Yolu	PV897541, PV902928, PV902898
***Megalocystis***	2597, 2698	*A. micracme* *	Hakkari/Çukurca	PV897524, PV902936, PV902906
	2627	*A. ermineus* *	Van/Gevaş	PV897531, PV902935, PV902905
	2734	*A. szowitsii* *	Ağrı/Doğubeyazıt	PV897542, PV902934, PV902904
***Poterion***	2519, 2538	*A. russelli* *	Ş.Urfa/Ceylanpınarı	PV897521, PV902938, PV902908
	2536	*A. russelli*	Ş.Urfa/Hilvan	
	2702	*A. brugueri*	Şırnak/Cizre	
	2705	*A. brugueri* *	Şırnak/Kumçatı	PV897522, PV902937, PV902907
	2710	*A. brugueri*	Şırnak/Nusaybin	
***Hymenostegis***	2589	*A. zohrabi*	Van/Erçek	
	2590	*A. zohrabi*	Ağrı/Gevaş	
	2626	*A. zohrabi* *	Van/Bahçesaray	PV897550, PV902955, PV902925
	2712	*A. velenowskyii* *	Van/Çaldıran	PV897546, PV902954, PV902924
	2632	*A. velenowskyii*	Van/Erciş	
	2591	*A. hymenocystis*	Van/Muradiye	
	2635	*A. hymenocystis* *	Van/Tendürek	PV897534, PV902948, PV902918
	2752, 2765	*A. hymenocystis*	Van/Gürpınar	
	2599, 2600	*A. hirticalyx*	Van/Hoşap	
	2601	*A. hirticalyx* *	Van/Erciş	PV897533, PV902949, PV902919
	2569	*A. lagopoides*	Van/Muradiye	
	2635	*A. lagopoides*	Van/Erciş	
	2576, 2581	*A. lagopoides*	Van/Bendimahi	
	2588	*A. lagopoides* *	Van/Altındere	PV897536, PV902946, PV902916
	2618	*A. lagopoides* *	Nevşehir/Ürgüp	PV897527, PV902945, PV902915
	2616	*A. lagopoides*	Van/Güzeldere	
	2622	*A. lagopoides*	Van/Yukarınarlı	
	2646	*A. lagopoides*	Ağrı/Çaldıran	
	2654	*A. lagopoides*	Van/Horasan	
	2761	*A. lagopoides*	Van/Sugeçer Köyü	
	2779	*A. lagopoides*	Sivas/Gürün	
	2783	*A. lagopoides*	Ağrı/Doğubeyazıt	
	2794	*A. lagopoides*	Ardahan	
	2798	*A. lagopoides*	Erzurum/Oltu	
	2584	*A. sosnowskii*	Van/Adaklı Köyü	
	2588, 2632, 2633	*A. sosnowskii*	Van/Erciş	
	2592	*A. sosnowskii* *	Van/Erçek	PV897540, PV902950, PV902920
	2593	*A. sosnowskii*	Van/Özalp	
	2660, 2667	*A. sosnowskii*	Erzurum/Oltu	
	2609, 2650	*A. trifoliastrum*	Van/Güzeldere	
	2781	*A. trifoliastrum* *	Van/Kurubaş	PV897543, PV902951, PV902921
	2652	*A. ciloensis* *	Van/Gürpınar	PV897529, PV902947, PV902917
	2527, 2575, 2622, 2659	*A. gueruenensis* *	Sivas/Gürün	PV897525, PV902956, PV902926
	2577, 2582, 2599, 2747	*A. uraniolimneus*	Van/Muradiye	
	2749, 2797	*A. uraniolimneus* *	Van/Hoşap	PV897544, PV902952, PV902922
***Macrophyllium***	2625	*A. dipodurus* *	Konya/Karasınır	PV897530, PV902940, PV902910
	2736	*A. dipodurus*	G.Antep/Arat Dağı	
	2620	*A. yukselii* *	Konya/Hadim	PV897548, PV902944, PV902914
	2542	*A. oleaefolius* *	Aksaray/Taptuk emre Köyü	PV897539, PV902943, PV902913
	2557	*A. oleaefolius*	Sivas/Şarkışla	
	2657	*A. oleaefolius*	Erzurum/İspir	
	2735	*A. oleaefolius*	G.Antep/Nizip	
	2738	*A. oleaefolius*	Sivas/Gemerek	
	2598	*A. longifolius*	Hakkari/Çukurca	
	2587, 2647, 2659, 2661	*A. longifolius* *	Van/Hasanabdal	PV897537, PV902942, PV902912
	2549	*A. isauricus* *	Konya/Taşkent	PV897535, PV902941, PV902911
	2670, 2793	*A. cephalotes* *	Artvin/Şavşat	PV897523, PV902939, PV902909
	2733, 2795	*A. cephalotes*	Artvin/Yusufeli	
***Hymenocoleus***	2672	*A. vaginans*	K.Maraş/Çağlayancerit	
	2776	*A. vaginans* *	Niğde/Ulukışla	PV897545, PV902953, PV902923
