# Supplementary material for: Molecular Insights into the Diversification and Biogeographic History of Six Astragalus L. Sections in the Turkish Flora
Source: Plants (Basel). 2025 Jul 18;14(14):2226. doi: 10.3390/plants14142226 (PMC12299922; doi:10.3390/plants14142226)
Supplement: Supplementary file 1 [file plants-14-02226-s001.zip › Supplementary Figure.pdf]

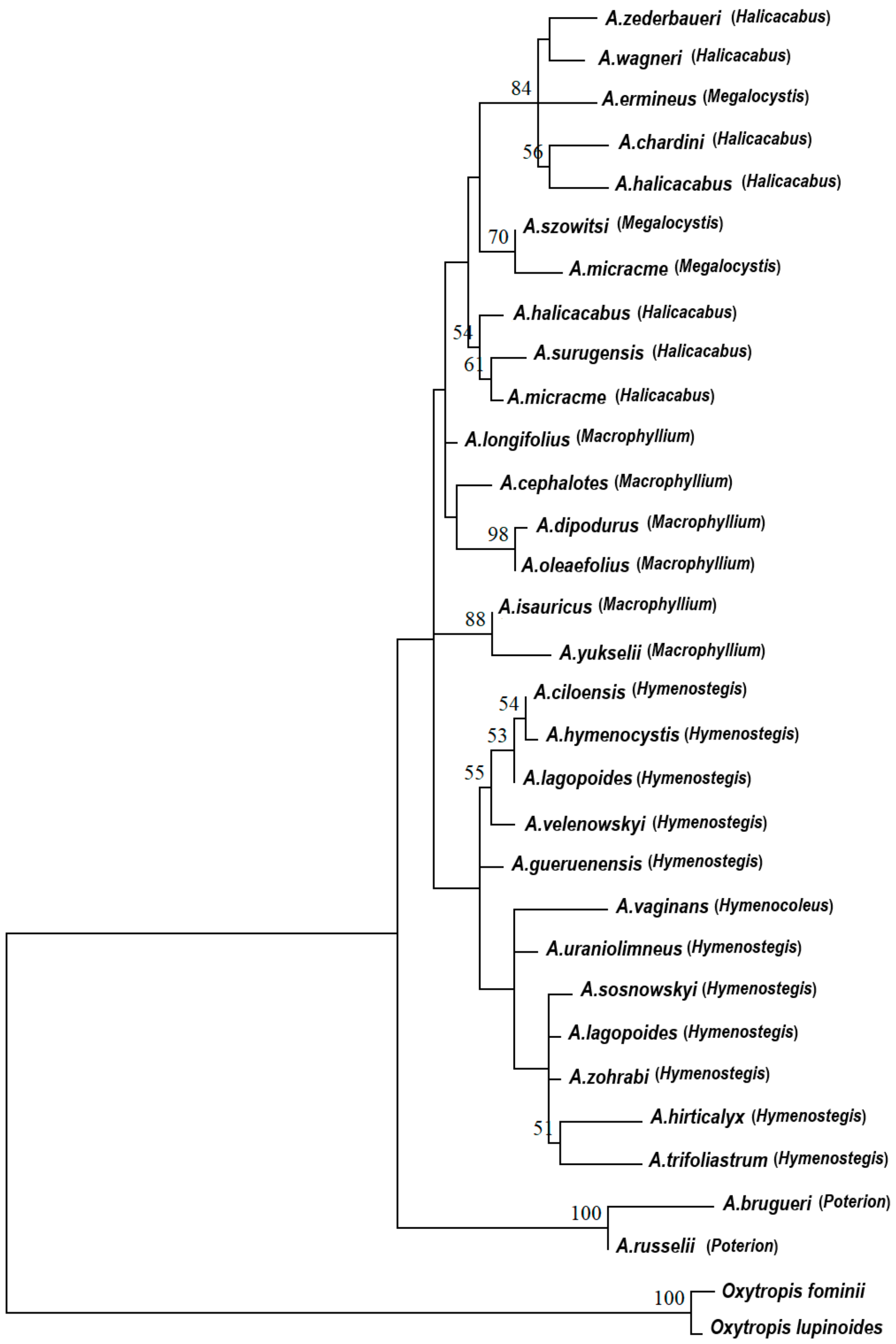

Figure S1. Phylogenetic tree with the GTR model with uniform distribution of combination of both cpDNA regions (*trnL5′*-L3′+ L3′-F(GAA)+*matK*) and nrDNA regions (ITS) of the studied species. The results of analysis with subsequent optimization (bootstrap values) are given next to the nodes (the bootstrap values lower than 50 are not shown).
