# Supplementary material for: Molecular Insights into the Diversification and Biogeographic History of Six Astragalus L. Sections in the Turkish Flora
Source: Plants (Basel). 2025 Jul 18;14(14):2226. doi: 10.3390/plants14142226 (PMC12299922; doi:10.3390/plants14142226)
Supplement: Supplementary file 1 [file plants-14-02226-s001.zip › Supplementary Tables.pdf]

**Table S1:** The number of base substitutions per site from averaging over all sequence pairs between species are shown. Analyses were conducted using the Maximum Composite Likelihood model by using combination of both cpDNA and nrDNA regions (*trn* L-F+*matK*+ITS)(Calculated by MEGA 11) (Overall mean genetic distance : 0.01).

| Species | 1 | 2 | 3 | 4 | 5 | 6 | 7 | 8 | 9 | 10 | 11 | 12 | 13 | 14 | 15 | 16 | 17 | 18 | 19 | 20 | 21 | 22 | 23 | 24 | 25 | 26 | 27 | 28 | 29 | 30 |
|---|---|---|---|---|---|---|---|---|---|---|---|---|---|---|---|---|---|---|---|---|---|---|---|---|---|---|---|---|---|---|
| *A.zederbaueri* | | | | | | | | | | | | | | | | | | | | | | | | | | | | | | |
| *A.surugensis* | 5,99E-03 | | | | | | | | | | | | | | | | | | | | | | | | | | | | | |
| *A.anthylloides* | 5,99E-03 | 1,70E-03 | | | | | | | | | | | | | | | | | | | | | | | | | | | | |
| *A.chardini* | 4,70E-03 | 7,28E-03 | 6,42E-03 | | | | | | | | | | | | | | | | | | | | | | | | | | | |
| *A.halicacabus* | 4,70E-03 | 7,27E-03 | 6,42E-03 | 4,27E-03 | | | | | | | | | | | | | | | | | | | | | | | | | | |
| *A.halicacabus* | 5,98E-03 | 2,56E-03 | 1,70E-03 | 6,42E-03 | 6,41E-03 | | | | | | | | | | | | | | | | | | | | | | | | | |
| *A.vagneri* | 2,98E-03 | 5,55E-03 | 4,70E-03 | 3,42E-03 | 4,27E-03 | 4,70E-03 | | | | | | | | | | | | | | | | | | | | | | | | |
| *A.szowitsi* | 5,56E-03 | 2,99E-03 | 2,99E-03 | 5,99E-03 | 5,99E-03 | 2,99E-03 | 5,13E-03 | | | | | | | | | | | | | | | | | | | | | | | |
| *A.ermineus* | 4,27E-03 | 5,13E-03 | 5,13E-03 | 4,71E-03 | 4,70E-03 | 5,13E-03 | 2,99E-03 | 4,70E-03 | | | | | | | | | | | | | | | | | | | | | | |
| *A.micracme* | 7,28E-03 | 4,70E-03 | 4,70E-03 | 6,86E-03 | 7,71E-03 | 4,70E-03 | 6,85E-03 | 1,70E-03 | 6,42E-03 | | | | | | | | | | | | | | | | | | | | | |
| *A.brugueri* | 1,73E-02 | 1,60E-02 | 1,52E-02 | 1,78E-02 | 1,87E-02 | 1,52E-02 | 1,56E-02 | 1,52E-02 | 1,78E-02 | 1,60E-02 | | | | | | | | | | | | | | | | | | | | |
| *A.russelii* | 1,34E-02 | 1,21E-02 | 1,12E-02 | 1,38E-02 | 1,47E-02 | 1,12E-02 | 1,16E-02 | 1,12E-02 | 1,38E-02 | 1,21E-02 | 3,86E-03 | | | | | | | | | | | | | | | | | | | |
| *A.cephalotes* | 6,41E-03 | 4,70E-03 | 3,84E-03 | 5,99E-03 | 6,84E-03 | 3,84E-03 | 5,13E-03 | 3,42E-03 | 6,42E-03 | 3,42E-03 | 1,34E-02 | 9,46E-03 | | | | | | | | | | | | | | | | | | |
| *A.dipodurus* | 8,14E-03 | 5,98E-03 | 5,13E-03 | 6,86E-03 | 8,57E-03 | 5,13E-03 | 6,85E-03 | 5,56E-03 | 8,14E-03 | 6,42E-03 | 1,65E-02 | 1,25E-02 | 3,84E-03 | | | | | | | | | | | | | | | | | |
| *A.isauricus* | 8,14E-03 | 4,70E-03 | 3,84E-03 | 6,85E-03 | 8,56E-03 | 3,84E-03 | 5,13E-03 | 5,13E-03 | 7,28E-03 | 6,85E-03 | 1,47E-02 | 1,08E-02 | 4,27E-03 | 5,55E-03 | | | | | | | | | | | | | | | | |
| *A.longifolius* | 5,98E-03 | 3,41E-03 | 2,56E-03 | 5,56E-03 | 6,41E-03 | 2,56E-03 | 4,70E-03 | 2,99E-03 | 5,99E-03 | 4,70E-03 | 1,38E-02 | 9,90E-03 | 2,13E-03 | 3,41E-03 | 2,98E-03 | | | | | | | | | | | | | | | |
| *A.oleaefolius* | 7,71E-03 | 5,56E-03 | 4,70E-03 | 6,43E-03 | 8,14E-03 | 4,70E-03 | 6,42E-03 | 5,13E-03 | 7,72E-03 | 5,99E-03 | 1,60E-02 | 1,21E-02 | 3,41E-03 | 4,25E-04 | 5,13E-03 | 2,98E-03 | | | | | | | | | | | | | | |
| *A.yukselii* | 9,43E-03 | 5,98E-03 | 5,13E-03 | 8,14E-03 | 9,86E-03 | 5,12E-03 | 6,41E-03 | 6,42E-03 | 8,57E-03 | 8,14E-03 | 1,60E-02 | 1,21E-02 | 5,55E-03 | 6,84E-03 | 2,13E-03 | 4,26E-03 | 6,41E-03 | | | | | | | | | | | | | |
| *A.lagopoides* | 8,56E-03 | 6,41E-03 | 5,55E-03 | 7,28E-03 | 8,99E-03 | 5,55E-03 | 7,27E-03 | 5,13E-03 | 8,57E-03 | 5,99E-03 | 1,51E-02 | 1,12E-02 | 5,12E-03 | 4,70E-03 | 5,12E-03 | 3,84E-03 | 4,27E-03 | 5,55E-03 | | | | | | | | | | | | |
| *A.lagopoides* | 7,27E-03 | 8,13E-03 | 7,27E-03 | 6,85E-03 | 7,70E-03 | 7,27E-03 | 5,98E-03 | 5,98E-03 | 7,28E-03 | 5,98E-03 | 1,60E-02 | 1,21E-02 | 5,12E-03 | 8,13E-03 | 6,84E-03 | 5,55E-03 | 7,70E-03 | 7,27E-03 | 3,41E-03 | | | | | | | | | | | |
| *A.ciloensis* | 8,13E-03 | 5,98E-03 | 5,12E-03 | 6,85E-03 | 8,56E-03 | 5,12E-03 | 6,84E-03 | 4,70E-03 | 8,14E-03 | 5,56E-03 | 1,56E-02 | 1,16E-02 | 4,69E-03 | 4,27E-03 | 5,55E-03 | 3,41E-03 | 3,84E-03 | 5,12E-03 | 4,25E-04 | 3,84E-03 | | | | | | | | | | |
| *A.hymenocystis* | 8,56E-03 | 6,41E-03 | 5,55E-03 | 7,28E-03 | 8,99E-03 | 5,55E-03 | 7,27E-03 | 5,13E-03 | 8,57E-03 | 5,99E-03 | 1,60E-02 | 1,21E-02 | 5,12E-03 | 4,70E-03 | 5,98E-03 | 3,84E-03 | 4,27E-03 | 5,55E-03 | 8,51E-04 | 4,26E-03 | 4,25E-04 | | | | | | | | | |
| *A.hirticalyx* | 9,44E-03 | 1,03E-02 | 9,01E-03 | 8,58E-03 | 9,86E-03 | 9,43E-03 | 8,15E-03 | 9,01E-03 | 9,45E-03 | 9,01E-03 | 1,87E-02 | 1,51E-02 | 7,28E-03 | 1,03E-02 | 9,86E-03 | 7,71E-03 | 9,87E-03 | 9,44E-03 | 7,28E-03 | 3,84E-03 | 6,85E-03 | 7,27E-03 | | | | | | | | |
| *A.sosnowskyi* | 7,70E-03 | 7,70E-03 | 6,84E-03 | 6,42E-03 | 8,13E-03 | 6,84E-03 | 6,41E-03 | 6,41E-03 | 7,71E-03 | 5,56E-03 | 1,56E-02 | 1,16E-02 | 4,69E-03 | 6,84E-03 | 6,40E-03 | 5,12E-03 | 6,41E-03 | 6,84E-03 | 2,98E-03 | 1,28E-03 | 3,41E-03 | 3,84E-03 | 4,27E-03 | | | | | | | |
| *A.trifoliastrum* | 9,00E-03 | 9,86E-03 | 9,00E-03 | 8,58E-03 | 9,42E-03 | 8,13E-03 | 7,71E-03 | 8,57E-03 | 9,01E-03 | 8,57E-03 | 1,87E-02 | 1,47E-02 | 6,84E-03 | 9,86E-03 | 9,43E-03 | 7,27E-03 | 9,43E-03 | 9,00E-03 | 7,27E-03 | 3,84E-03 | 6,84E-03 | 7,27E-03 | 5,99E-03 | 4,27E-03 | | | | | | |
| *A.uraniolimneus* | 7,27E-03 | 7,27E-03 | 6,41E-03 | 6,85E-03 | 7,70E-03 | 6,41E-03 | 5,99E-03 | 5,98E-03 | 7,28E-03 | 7,70E-03 | 1,60E-02 | 1,21E-02 | 5,98E-03 | 7,27E-03 | 5,98E-03 | 4,69E-03 | 6,84E-03 | 6,41E-03 | 2,55E-03 | 1,70E-03 | 2,98E-03 | 2,56E-03 | 5,56E-03 | 2,98E-03 | 5,55E-03 | | | | | |
| *A.vaginans* | 8,14E-03 | 7,27E-03 | 7,28E-03 | 7,72E-03 | 8,57E-03 | 7,27E-03 | 6,85E-03 | 6,85E-03 | 7,28E-03 | 8,57E-03 | 1,65E-02 | 1,25E-02 | 6,84E-03 | 8,13E-03 | 6,84E-03 | 5,55E-03 | 7,70E-03 | 7,27E-03 | 5,98E-03 | 5,13E-03 | 6,41E-03 | 6,84E-03 | 7,28E-03 | 5,55E-03 | 6,85E-03 | 4,27E-03 | | | | |
| *A.velenowskyi* | 7,70E-03 | 6,41E-03 | 5,55E-03 | 8,14E-03 | 8,99E-03 | 5,55E-03 | 6,41E-03 | 5,99E-03 | 8,57E-03 | 7,71E-03 | 1,43E-02 | 1,03E-02 | 5,12E-03 | 5,55E-03 | 5,12E-03 | 3,83E-03 | 5,13E-03 | 5,55E-03 | 1,70E-03 | 4,26E-03 | 2,13E-03 | 1,70E-03 | 7,28E-03 | 3,83E-03 | 7,27E-03 | 2,56E-03 | 5,98E-03 | | | |
| *A.zohrabi* | 7,27E-03 | 8,13E-03 | 7,27E-03 | 6,85E-03 | 7,70E-03 | 7,27E-03 | 5,98E-03 | 6,84E-03 | 7,28E-03 | 6,84E-03 | 1,60E-02 | 1,21E-02 | 5,12E-03 | 8,13E-03 | 6,83E-03 | 5,55E-03 | 7,70E-03 | 7,27E-03 | 4,26E-03 | 8,50E-04 | 4,69E-03 | 4,27E-03 | 3,84E-03 | 1,28E-03 | 3,84E-03 | 1,70E-03 | 5,12E-03 | 3,41E-03 | | |
| *A.gueruenensis* | 7,70E-03 | 5,98E-03 | 5,13E-03 | 7,28E-03 | 8,13E-03 | 5,12E-03 | 6,41E-03 | 4,70E-03 | 7,71E-03 | 5,56E-03 | 1,30E-02 | 9,03E-03 | 2,98E-03 | 5,98E-03 | 4,69E-03 | 3,41E-03 | 5,56E-03 | 5,13E-03 | 2,13E-03 | 2,98E-03 | 2,56E-03 | 2,98E-03 | 5,99E-03 | 2,55E-03 | 5,98E-03 | 2,98E-03 | 5,55E-03 | 2,13E-03 | 2,98E-03 | |

**Table S2:** The number of base substitutions per site from averaging over all sequence pairs between sections are shown. Analyses were conducted using the Maximum Composite Likelihood model by using both cpDNA and nrDNA regions (*trn* L-F+*mat*K+ITS) (Calculated by MEGA 11).

| **Sections** | **1** | **2** | **3** | **4** | **5** | **6** |
|---|---|---|---|---|---|---|
| ***Halicacabus*** | | | | | | |
| ***Megalocystis*** | 5.07E-03 | | | | | |
| ***Poterion*** | 1.46E-02 | 1.44E-02 | | | | |
| ***Macrophyllium*** | 5.94E-03 | 6.02E-03 | 1.31E-02 | | | |
| ***Hymenostegis*** | 7.35E-03 | 7.04E-03 | 1.39E-02 | 6.12E-03 | | |
| ***Hymenocoleus*** | 7.58E-03 | 7.57E-03 | 1.45E-02 | 7.06E-03 | 5.91E-03 | |
